# When Public Health Research Meets Social Media: Knowledge Mapping From 2000 to 2018

**DOI:** 10.2196/17582

**Published:** 2020-08-13

**Authors:** Yan Zhang, Bolin Cao, Yifan Wang, Tai-Quan Peng, Xiaohua Wang

**Affiliations:** 1 School of Media and Communication Shenzhen University Shenzhen China; 2 Department of Communication Michigan State University East Lansing, MI United States

**Keywords:** social media, public health, infodemiology, infoveillance, topic modeling, research theme, research method

## Abstract

**Background:**

Social media has substantially changed how people confront health issues. However, a comprehensive understanding of how social media has altered the foci and methods in public health research remains lacking.

**Objective:**

This study aims to examine research themes, the role of social media, and research methods in social media–based public health research published from 2000 to 2018.

**Methods:**

A dataset of 3419 valid studies was developed by searching a list of relevant keywords in the Web of Science and PubMed databases. In addition, this study employs an unsupervised text-mining technique and topic modeling to extract research themes of the published studies. Moreover, the role of social media and research methods adopted in those studies were analyzed.

**Results:**

This study identifies 25 research themes, covering different diseases, various population groups, physical and mental health, and other significant issues. Social media assumes two major roles in public health research: produce substantial research interest for public health research and furnish a research context for public health research. Social media provides substantial research interest for public health research when used for health intervention, human-computer interaction, as a platform of social influence, and for disease surveillance, risk assessment, or prevention. Social media acts as a research context for public health research when it is mere reference, used as a platform to recruit participants, and as a platform for data collection. While both qualitative and quantitative methods are frequently used in this emerging area, cutting edge computational methods play a marginal role.

**Conclusions:**

Social media enables scholars to study new phenomena and propose new research questions in public health research. Meanwhile, the methodological potential of social media in public health research needs to be further explored.

## Introduction

Social media has deeply penetrated people’s lives in many aspects. In developing and developed societies, social media has played a significant role in health management and disease control [1]. Social media is integrated into empirical examinations of the prevention and control of various types of diseases, including emerging, infectious, and chronic diseases [2-4]. Social media has been employed to study health phenomena among different populations or social groups such as children, pregnant women, and older adults [5,6]; different genders [7,8]; and individuals in various social classes [9].

Agencies widely use social media to fulfill different health purposes. For the general public, social media is used to satisfy its orientation for health information, linking with health services and communication with others who share the same health interests [10]. For public health professionals and organizations, social media (eg, Facebook, Grindr, mobile apps) serves as a multifunctional tool to launch interventions to reach a wide array of the population efficiently [11-14]. The large volume of mobility and discourse data on social media (eg, Twitter) can be conducive for public health management, including disease surveillance, assessment, and control [15-17].

These studies have demonstrated the increase of scholarly interest in empirical research conducted on social media platforms with public health goals, including the social media–based public health research in this study [18-21]. Although many scholars in social science and public health have contributed to this field, the overview about how social media has been integrated into public health research remains limited. Prior systematic reviews on social media–based public health research often focused on certain domains or topics. Many reviews systematically investigated the effectiveness of social media interventions for varied specific health outcomes, such as the promotion of safe sexual health behaviors [22], vaccine uptake [23], noncommunicable disease management [24], and HIV prevention [19]. Scholars are often dedicated to one or two domains, neglecting the fact that using social media in one field may shed light on another. Meanwhile, focusing on one particular area, these reviews often face challenges to identify similar patterns across domains and capture an integrated picture about social media–based public health research. In addition, most existing reviews included a limited number of original articles [25,26]. Even a review of systematic reviews only extracted few studies [18]. In this emerging and fast-growing subject field, the limited literature being included may fail to provide a panoramic description of social media–based public health research.

Furthermore, most prior systematic reviews have adopted a top-down approach and therefore may have narrowed the view by overlooking certain nuances and novelties that have been emerging. This study adopts a bottom-up approach [27] to understand the growth of social media–based public health research and remain open to map the intellectual landscape in this area. Specifically, the study aims to address the following research questions:

- RQ 1: What are the major publication trends of social media–based public health research since 2000?
- RQ 2: What are the major research themes in social media–based public health research since 2000?
- RQ 3: What role does social media play in social media–based public health research since 2000?
- RQ 4: What are the major research methods adopted in social media–based public health research since 2000?

## Methods

### Data Collection

**Figure 1:**
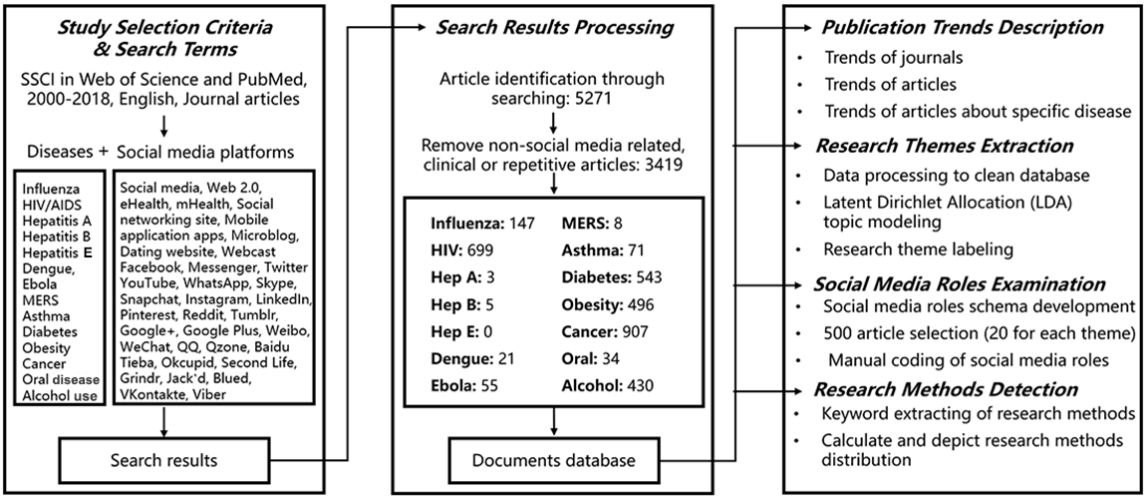
Knowledge-mapping workflow of social media–based public health research from 2000 to 2018.

To examine how social media has been adopted and integrated into public health research, a list of terms was identified and the Web of Science (Clarivate Analytics) and PubMed databases were searched (see Figure 1). Lists of keywords about social media and disease were established. This study focused on emerging, infectious, and chronic diseases. Specifically, 14 diseases were selected that are of high prevalence among the population or pose major public health threats according to the World Health Organization [28]: influenza, HIV, hepatitis A, hepatitis B, hepatitis E, dengue, Ebola, Middle East respiratory syndrome (MERS), asthma, diabetes, obesity, cancer, oral disease, and alcohol use. A list of keywords was constructed. Then a list of social media keywords, including the general social media categories and specific social media platforms, was established.

The lists of disease and social media keywords were combined pairwise and submitted to search titles, abstracts, and keywords of published studies in the databases of the Web of Science and PubMed. The publication period was limited to between 2000 and 2018. The article language was limited to English, and document type was limited to scholarly journal articles. Considering the PubMed database includes numerous medical and clinical studies that are beyond the research scope of this study, the search results were refined by setting the broad subject terms as related categories, such as public health and medical informatics to reduce the noise in the search results. This search strategy led to the identification of 5271 articles in the two databases. Then, another round of data checking to remove unqualified studies such as non–social media–related articles, duplicate records, and clinical studies was implemented. Finally, a dataset of 3419 articles was collected for further analysis. Ultimately, document level information of the 3419 articles from the databases, including authors, article title, journal title, abstract, author keywords, and cited references, was retrieved.

### Data Analysis

An automatic text-mining approach was adopted to extract research themes in the field of social media–based public health research. As the abstracts of published studies conveyed the themes or foci of the articles [29], the article abstracts were mined through latent Dirichlet allocation (LDA) topic modeling, which is a popular unsupervised text-mining technique in computational social science. LDA topic modeling helps recognize the structure of research development, current trends, and interdisciplinary landscapes of research [27]. The LDA topic modeling [30] was implemented with the tm package in the R software (R Foundation for Statistical Computing). Data preprocessing, such as removal of stop words and numbers, was performed before the LDA topic modeling.

Numerous LDA topic models were estimated with various numbers of topics. These models were evaluated on the basis of three main criteria: (1) a substantial proportion of articles exists under each topic, (2) themes show independence with one another and the lists of top terms of topics are not highly overlapped or not relevant, (3) models differing in theme number are compared to identify the nuanced differences and determine the best theme extraction by assuring that each term list is coherent. Finally, a topic model with 25 topics, which presented adequate discrimination between topics and convergence within a topic, was selected. The articles were classified into the research theme with which they had the greatest probability scores.

To understand how social media has been integrated into public health research with different thematic foci, a manual content analysis was conducted among a randomly selected sample of 500 abstracts (20 from articles in each theme) to understand the role of social media. A coding scheme was developed by two authors of this study. The two authors first separately coded a subsample of 60 randomly selected abstracts to construct the coding scheme. After several rounds of exploration and discussion, they achieved a satisfying intercoder reliability (as measured with a Cohen kappa). The role of social media is categorized into two main types. First, social media provides a substantial research interest for public health research, which includes the use of social media for intervention, as human-computer interaction characteristics, as platforms of social influence, and for risk assessment or disease prevention. Second, social media is employed as a context in public health research with social media as mere reference, as participant recruitment tool, or as data source. Categories and their definitions are further illustrated in the analytical findings section (also see Multimedia Appendix 1).

Finally, the research methods adopted by social media–based public health research were identified by searching a list of keywords associated with various research methods among the titles, abstracts, and keywords of the retrieved studies. Figure 1 summarizes the study workflow.

## Results

### Publication Trends in Social Media–based Public Health Research

Publication trends in the field of social media–based public health research in the past two decades were presented in 3 dimensions: growth of overall publications, growth of publications by specific diseases, and growth of journal outlets.

**Figure 2:**
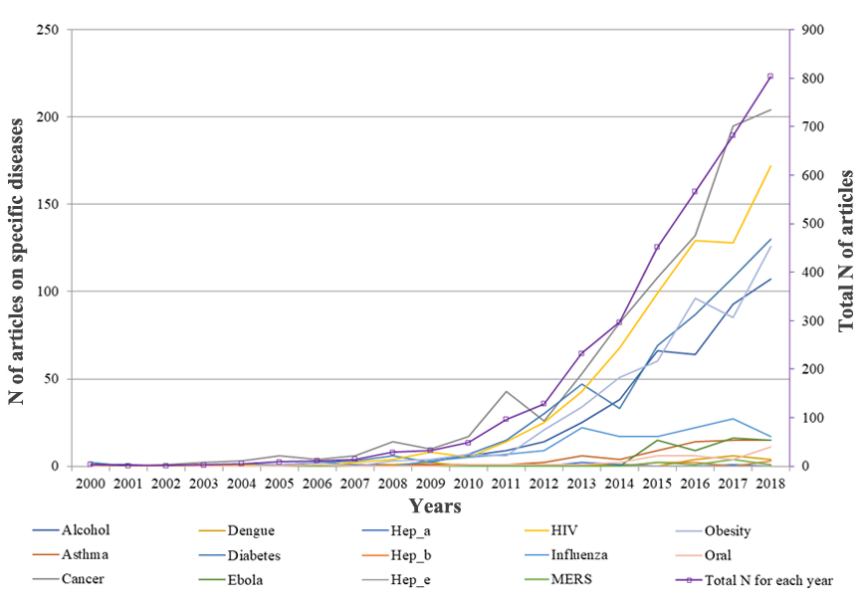
Total number of articles for each disease in the Social Sciences Citation Index from 2000 to 2018.

Empirical studies in this area were relatively limited in the first decade (2000 to 2010), which demonstrated a minimal increase as shown in Figure 2. A significant annual increase was observed from 2011 to 2018. Such trends intersected with the advancement of the internet, especially that of social media. Although popular social media platforms, such as Facebook, Twitter, and Instagram, were launched before 2010, they have been widely accepted worldwide since 2010. This implies that social media–based public health research is a study area responsive to technological development. Prior research also demonstrated similar findings that internet research evolved along with technological development [29].

Social media has been increasingly incorporated into the studies of certain types of diseases in the past decades. Dramatic increases in research occurred on cancer, HIV, diabetes, obesity, and alcohol use after 2010. Other diseases, such as influenza, hepatitis A, hepatitis B, dengue, Ebola, MERS, asthma, and oral disease, showed a relatively slow growth rate that remained quite stable from 2000 to 2018.

**Figure 3:**
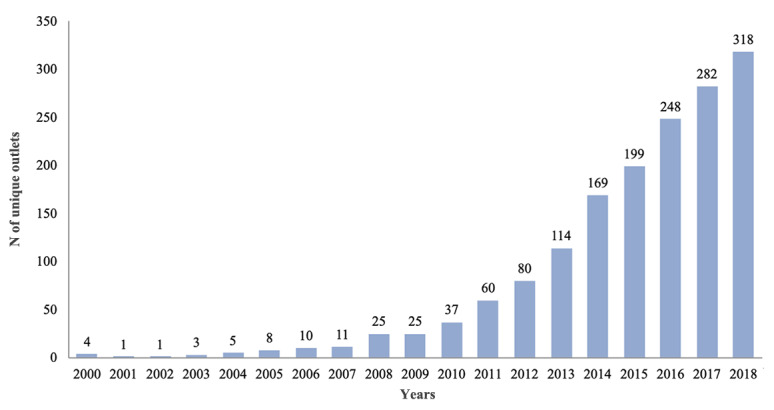
Number of unique outlets that published output of social media–based public health research from 2000 to 2018.

For the journal outlets, a total of 799 journals published studies in these areas (see Figure 3). Table 1 reports the 15 most visible journals in this area. Among them, Journal of Medical Internet Research, which published 331 articles, is the most visible one accounting for 9.68% of the total publications. Moreover, JMIR sister journals, such as JMIR mHealth and uHealth (165 publications), JMIR Research Protocols (114 publications), and JMIR Public Health and Surveillance (49 publications) also showed great interest in this domain. Other high-ranked journals included PLoS One, BMC Public Health, Studies in Health Technology and Informatics, AIDS and Behavior, and BMJ Open, each occupy more than 1.5% of publication in this field.

**Table 1:** Top journals in social media–based public health research.

Number	Journal name	Publications, n (%)
1	Journal of Medical Internet Research	331 (9.7)
2	JMIR mHealth and uHealth	165 (4.8)
3	PLoS One	123 (3.6)
4	JMIR Research Protocols	114 (3.3)
5	BMC Public Health	72 (2.1)
6	Studies in Health Technology and Informatics	68 (2.0)
7	AIDS and Behavior	53 (1.5)
8	BMJ Open	52 (1.5)
9	JMIR Public Health and Surveillance	49 (1.4)
10	Journal of Health Communication	49 (1.4)
11	International Journal of Medical Informatics	37 (1.1)
12	Journal of Diabetes Science and Technology	37 (1.1)
13	Health Communication	34 (1.0)
14	Computers in Human Behavior	33 (1.0)
15	International Journal of Environmental Research and Public Health	31 (0.9)

### Research Themes in Social Media–Based Public Health Research

The 25 extracted research themes were labeled on the basis of the top 15 most frequently used terms associated with each theme and the articles assigned to the theme. Table 2 presents the lists of terms under each theme. A network graph of theme-word probability of the 25 research themes is provided in Multimedia Appendix 2. Multimedia Appendix 3 displays a typical study under each theme.

The percentages in Table 2 reveal the article distribution across research themes. The articles under each theme varied greatly from 2.22% (76/3419) to 7.93% (271/3419; Table 2), with men and HIV occupying the largest number of articles and reproductive cancers the least. Among them, the mHealth family, the themes about mHealth (themes 1, 2, 3, 4), contained a large body of 653 articles. Themes about substance use (themes 6, 7, 8) comprised 409 articles. Another big cluster was cancer (themes 10, 12, 13), which consisted of 385 articles.

The 25 research themes were further grouped into 6 research clusters on the basis of similar concerns and associations. The first cluster was on health education, which comprises 4 themes: health education–school and students, health education–family and oral/dental health, mHealth and medical decisions, and pregnancy. Health education aims to prevent diseases through improving people’s knowledge and health efficacy. School and family, as the main scenes for the students to learn health beliefs and behaviors, have been the foci of health education. Sexual health education on condom use and pregnancy have also attracted increasing scholarly attention.

The second cluster was on health management with the help of mHealth. The themes mHealth and weight control, mHealth and diabetes management, digital campaigns in targeted populations, social media and alcohol drinking, substance usage and cessation, food and asthma, and vaccination and immunization all fell into this cluster. This indicates the functional attributes of social media to help manage health problems. Social media use is used to intervene in certain unhealthy behaviors and promote healthy behavior adoption.

The third cluster, cancer studies, includes women’s cancer, reproductive cancer, cancer survivor, and caregiving on social media. Cancer is one of the world’s largest health problems and a significant cause of death. Thus, continuous attention has been paid to cancer studies.

The fourth cluster, infectious diseases, includes HIV as a key topic: mHealth and HIV; men and HIV; and infectious disease, health campaign, and stigma belong to this cluster. In this line of research, social media provides breakthrough channels to reach risky subgroups and focuses more attention on campaigns to reduce the stigma surrounding infectious diseases.

The fifth cluster was on mental health issues. This cluster consists of two themes: mental health and substance use and mental health–depression and digital technology. Mental health problems have been prominent in modern society. Digital technology is considered a cause and a solution to mental health issues.

The sixth cluster was on extended health research empowered by social media: health and human mobility, health marketing, surveillance and Twitter, and eHealth–miscellaneous. These research areas have flourished due to the availability of geographical information, mass user behavioral data, and extensive online discourse on social media platforms.

**Table 2:** Research themes and the top 15 keywords under each theme.

Number	Research themes	Top 15 keywords	n (%)
1	mHealth and weight management	App, weight, loss, selfmonitor, usabl, mHealth, adher, exercise, download, dietary, BMI, Fit, Android, coach, mainten	208 (6.08)
2	mHealth and diabetes management	diabet, selfmanag, glucos, usabl, mHealth, adolesc, HbA_1c_, TDM, young, selfcar, older, glycem, insulin, selfefficacy, cardiovascular	192 (5.62)
3	mHealth and medical decisions	decis, mHealth, intent, peer, consum, screen, trust, cell, doctor, choic, privacy, navig, leader, read, worker	103 (3.01)
4	mHealth and HIV	adher, mhealth, HIV, literacy, SMS, ART, portal, selfmanag, PLWH, retent, digit, RCTs, beta, viral, nurs	150 (4.39)
5	Men and HIV	HIV, men, sexual, MSM, partner, PrEP, AOR, gay, condom, drug, young, STI, YMSM, websit, Latino	271 (7.93)
6	Alcohol drinking and social media	alcohol, drink, young, consumpt, student, Facebook, post, colleg, alcoholrel, SNS, exposur, norm, adolesc, market, peer	174 (5.09)
7	Substance use and cessation	smoke, cessat, smoker, drug, tobacco, quit, marijuana, cigarett, EMA, abstin, substanc, addict, ecolog, alcohol, momentary	115 (3.36)
8	Mental health and substance use	symptom, mental, substanc, pain, disord, veteran, drug, cope, distress, adolesc, tan, fatigu, abus, stress, pro	120 (3.51)
9	Mental health–depression and digital technologies	depress, pain, digit, anxiety, adolesc, symptom, suicid, cancer, disord, older, memory, genet, injury, dengu, young	112 (3.28)
10	Women’s cancer	cancer, breast, screen, women, vaccin, HPV, campaign, prostat, colorect, cervic, lung, imag, news, cancerrel, papillomavirus	151 (4.42)
11	Pregnancy	women, pregnanc, pregnant, mother, gestat, GDM, matern, worker, CHWS, child, contracept, HCV, mHealth, postpartum, antenat	117 (3.42)
12	Reproductive cancers	cancer, ovarian, prostat, gene, polymorph, cultur, genotyp, postop, cohort, predict, nutrit, genet, PON, BRCA, surgic	76 (2.22)
13	Cancer survivor care	cancer, survivor, emot, breast, psychosoci, oncolog, young, modul, wellb, survivorship, AYA, selfmanag, QOL, depress, consult	158 (4.62)
14	Caregiving on social media	Facebook, post, caregiv, page, blog, comment, emot, CRC, virtual, channel, Twitter, friend, profil, chat, fit	136 (3.98)
15	Vaccination and immunization	vaccin, influenza, predict, coverag, season, event, news, queri, flu, immun, volum, forecast, surveil, websit, outbreak	119 (3.48)
16	Infectious disease, health campaigns and stigma	Ebola, HIV/AIDS, campaign, epidem, stigma, outbreak, audienc, Africa, outreach, facil, news, post, neighbourhood, stori, IBD	110 (3.22)
17	Food and asthma	food, asthma, nutrit, game, children, intak, consumpt, dietari, exposur, veget, infant, eat, weight, beverag, feed	146 (4.27)
18	Health education–family and oral/dental health	parent, websit, children, oral, dental, child, readabl, read, grade, instrument, discern, childhood, rank, pediatr, page	128 (3.74)
19	Health education–school and students	student, nurs, physician, mHealth, skin, school, melanoma, sun, cluster, EMR, hypertens, rural, India, NCDS, CVD	107 (3.13)
20	Health and human mobility	map, street, neighborhood, urban, walk, audit, built, sale, resid, agreement, happi, crowdsourc, hookah, LOS, imag	111 (3.25)
21	Digital campaigns in targeted populations	youth, advertis, rural, Hispan, adolesc, percent, young, homeless, campaign, urban, digit, women, black, cultur, underserv	131 (3.83)
22	Health behavior guidelines	behavior, guidelin, usag, practition, programm, ethic, sedentary, men, websit, GPS, ICT, screen, kingdom, citat, geosoci	86 (2.52)
23	Health marketing and social media	video, YouTub, market, brand, product, girl, adolesc, tobacco, industry, consum, company, ecigarett, boy, cigarett, surgery	100 (2.92)
24	Surveillance and Twitter	tweet, Twitter, surveil, post, sentiment, ILI, influenza, outbreak, detect, drug, opinion, mention, retweet, marijuana, pandem	165 (4.83)
25	eHealth–miscellaneous	eHealth, phase, eat, referr, client, mHealth, telemedicin, uncertainty, COPD, reward, PLHIV, emot, EVD, static, compet	133 (3.89)

### Roles of Social Media in Public Health Research

#### Social Media as Research Context or Substantial Interest

Social media is integrated into public health research by providing a new research context or producing new substantial interest in public health research.

When social media was adopted as a research context, social media was specifically considered as a mere reference, a platform for participant recruitment, and as a data source. When social media was adopted as a mere reference, research mostly used social media as a tool to offer intervention and facilitate the health management of individuals. For the role of a platform for recruitment, research either recruited participants through distributing questionnaires or posting participant recruitment announcements on social media (eg, Facebook) or employed users of certain social media platforms as the study target group (Grindr for the men who have sex with men group). For the role of data source, social media could contribute to collecting data in text, image, video, and app interface formats and collecting published posts and articles for meta-analysis or scope review.

When social media produced substantial interests for public health research, social media was used for intervention; employed to study human-computer interaction characteristics; used as a platform of social influence; and used for disease surveillance, risk assessment, or prevention. Under these 4 broad categories, the role of social media is described as follows:

#### Intervention

For public health intervention, the 4 subroles of social media in the published studies are as follows: (1) interactive intervention tool targeted at changing personal and environmental risky health factors, (2) intervention information-distributing tool (1-way and not real-time interactive), (3) source for health information seeking, such as YouTube and other platforms, and (4) usability test of social media platforms as intervention instruments.

#### Human-Computer Interaction Characteristics

Under this role, social media was used to serve the goal of revealing (1) the public’s attitudes toward technology and social media for health use, (2) characteristics and behaviors of social media users and groups, (3) factors affecting the health behaviors or attitudes of users on social media, and (4) consequences/influences on health behaviors caused by (popular) social media.

#### Platform of Social Influence

Social networking and interaction between different individuals and groups on social media could facilitate the change of health behaviors through the following approaches: (1) building online (support) groups for patients, such as cancer patient groups on Facebook, (2) promoting physician-patient communication or information seeker–provider communication, (3) enhancing health-related marketing, such as precision advertising, and (4) changing public health behavior at a macro level. All of these approaches are representations of social influence in online communities.

#### Disease Surveillance, Risk Assessment, or Prevention

The digital traces of online behaviors and massive online discourse granted opportunities to understand health conditions at a population level. For instance, Google trends and search query records could grant references to predict the possibility of a flu outbreak at an early stage.

**Figure 4:**
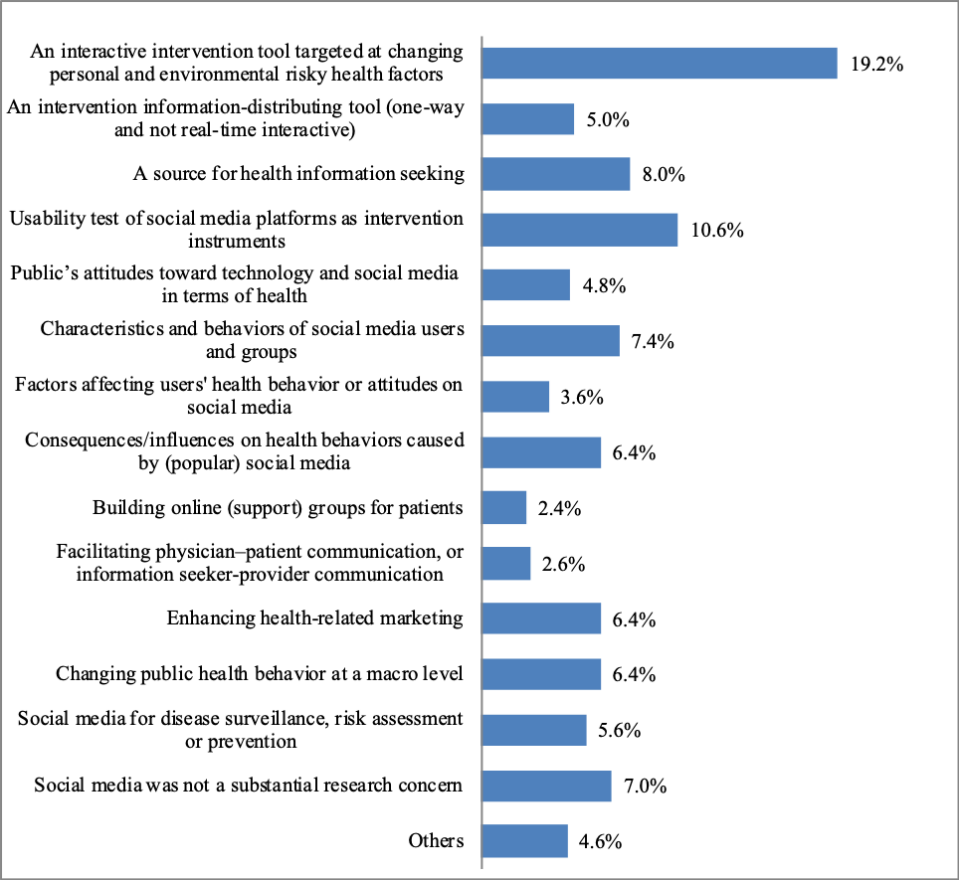
Article distributions based on social media as substantial interest.

Figure 4 presents the percentages of articles in each type of social media role. The results showed that among substantial interest, “an interactive intervention tool targeted at changing personal and environmental risky health factors” accounted for the largest percentage of 19.2%, followed by “usability test of social media platforms as intervention instruments” (10.6%), and “a source for health information seeking” (8.0%). Four of other types, “characteristics and behaviors of social media users and groups” (7.4%), “consequences/influences on health behaviors caused by (popular) social media” (6.4%), “enhancing health-related marketing” (6.4%), and “changing public health behavior as macro influence” (6.4%), also occupied a relatively larger proportion of more than 6%.

**Figure 5:**
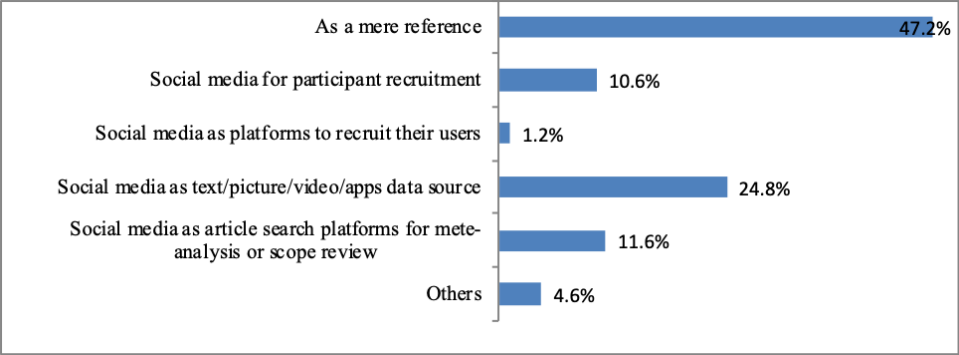
Article distributions based on social media as research context.

Among the dimension of social media as research context (Figure 5), “as a mere reference,” which took social media as a research background or a research environment, played the dominant role (47.2%). The second most frequent role that social media plays was content data source (ie, text/picture/video/app data sources, 24.8%). The other three were “social media as article search platform for meta-analysis or literature review” (11.6%), “for participant recruitment” (10.6%), and “as platforms to recruit their users” (1.2%).

### Research Methods in Social Media–Based Public Health Research

Public health research with social media data was dominated by traditional quantitative research methods, whereas cutting edge computational methods played a minor role. Among all the articles, 30.6% employed survey method, 24.0% employed experiment design, 22.7% employed qualitative methods (eg, field observation, in-depth interview, and focus group), 8.3% included employed digital methods (including digital tracks analysis and computational methods, such as text mining, sentiment analysis, agent-based modeling, and network modeling), and 5.6% employed traditional content analysis.

**Figure 6:**
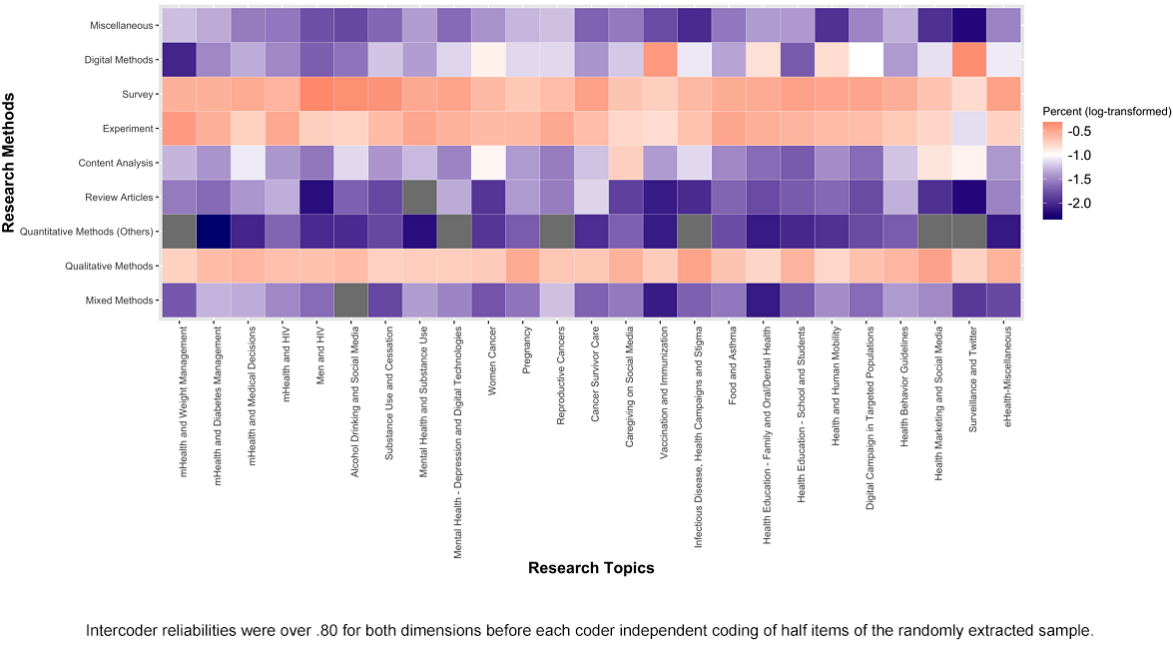
Research methods adopted under research topics.

Figure 6 demonstrates that the method distributions under the 25 research themes were similar to the general distribution among the whole body of the studies. Survey and experiment were the two most adopted methods, whereas review article number was relatively small among all themes.

## Discussion

### Principal Findings

With a bottom-up approach, this study provided a panoramic mapping of the landscape of social media–based public health research. By analyzing publication trends, research themes, roles of social media, and research methods adopted in this emerging research area, this study concluded that (1) social media has penetrated almost all the health-related processes and domains since 2010, showing a dramatic increase in the research body; (2) existing social media–based public health research mainly focuses on 25 themes in 6 clusters; (3) social media generally played two roles in public health research: generating substantial research interest and providing a research context/platform; (4) existing social media–based public health research is dominated by traditional research methods while the share of computational method is on the rise. The panoramic mapping can help scholars understand the state of the art in this research area and what is under- or overstudied in this field. This study can enable scholars across various disciplines to understand each other’s needs and contribute jointly to health promotion and disease control. Here, three notable issues that possess theoretical and methodological implications in social media–based public health research are elaborated.

### When Public Health Research Meets Social Media: From New Phenomena to New Questions

Social media has infiltrated almost all health-related processes and domains with the rapid advancement of social and mobile media. This dramatic change has entailed many new phenomena to be explored in public health research. The findings of this study are consistent with previous studies that found that almost one-third of internet studies have focused on eHealth and mHealth since 2009 [29] and a trend toward digitization exists in health care [31]. Many traditional public health activities, such as health education, health promotion, and disease surveillance, have taken advantage of social media technologies to become digitized [32-34]. Social media has substantially altered how individuals seek and share health information, discuss health issues, and engage in health behaviors [35]. Social media also provides innovative ways to change health behaviors in various domains, such as smoking cessation, substance use, weight control, HIV prevention, and cancer screening [36-38]. Consistent with previous reviews on social media and public health studies, this study concludes that social media contributes to these public health domains by broadening the reach of health education, providing accessible online professional consultation, and improving the efficacy of access to care and medication uptake, etc [19,39]. Moreover, an upward trend of integrating social media in various public health campaigns exists due to the instrumental benefits of social media technologies, such as lower intervention cost, higher user engagement, higher efficiency, and better documentation of the process [40].

When public health research meets social media, new topics have emerged and attracted the attention of public health scholars [41]: mHealth and social media–empowered health research. “Digital campaigns in targeted populations” and “surveillance and Twitter” are typical new topics where researchers frequently examine new research questions [42,43]. For example, researchers discuss how to employ user-generated content together with geolocation information to predict an outbreak of an emerging disease or visually map their diffusion routes and locate the risky population [44]. The digital trace on social and mobile media offers many possibilities to study online health behaviors such as online health information–seeking, online social support, and online medical consultation behaviors [45]. In addition, some health topics have attracted burgeoning attention in the era of social media. For instance, mental health problems have been identified as significant concerns among the 25 themes in this study. However, no conclusion has yet been reached whether and how the adoption and use of social media alleviates or exacerbates mental health problems [46].

This relatively new domain calls for in-depth exploration. New phenomena and new questions raised by social media are of practical and theoretical significance for public health research. Timely responses to those new phenomena via scientific research can promote the advancement of the domain to keep up with technology advances and establish a realistic understanding of what social media can and cannot do in public health. Meanwhile, public health research should delve into scientific research questions behind those new phenomena and address them either by exploiting the existing body of knowledge or exploring new methods and knowledge to extend the domain.

### When Public Health Research Meets Social Media: Methodological Potential to Be Further Tapped

When public health research meets social media, the dominant research methods are traditional quantitative methods, despite the growing interest in computational methods among public health scholars. The methodological potentials of social media for public health research can and should be further tapped. Specifically, the potential of social media in participant recruitment and measurement development has direct and salient implications to public health research.

Social media substantially facilitates participant recruitment in public health research. Recruiting research participants from specific groups of individuals who have sensitive health issues or are stigmatized in society, such as people living with HIV/AIDS or individuals with mental health issues, remains a significant challenge for public health scholars [19,47]. Given the size and heterogeneity of social media users, recruiting a fairly sizable number of subjects from particular social groups to participate in public health surveys and experiments should be possible. More importantly, participants recruited from online platforms such as Facebook and Amazon’s Mechanical Turk can have significant heterogeneity in their demographic characteristics (eg, age, gender, race, cultural background) and other key variables relevant to specific researcher contexts [48]. Nevertheless, it is worth noting here that the representativeness of participants recruited on social media needs to be empirically evaluated in particular contexts. Amazon’s Mechanical Turk workers are not a generalizable population with regard to health status and behaviors in the United States [49]. Without an empirical evaluation of representativeness of recruited subjects on social media, researchers should be cautious in the generalizability of their research findings. Moreover, ethical issues involved in participant recruitment via social media platforms have become more prominent and challenging. Due to the anonymity of social media users, it is extremely difficult if not impossible to obtain informed consent beforehand from recruited participants. When users of a social media platform accept the terms of service of the platform, can researchers assume that the users have given an explicit or implicit consent to participate in any type of experiment or intervention conducted on the platform [50]? We do not have a widely accepted ethical guideline in this regard. A collective effort from the scientific community is needed to outline responsible and ethical conduct in this emerging research area.

Social media contributes to public health research by providing refreshed measurements of existing concepts or new observations of emerging phenomena. Rich semantic information in digital traces can provide a social telescope [51] with which to observe or infer what health information is produced, shared, and consumed by ordinary users. Multiple social and interactive relations in digital traces facilitate empirical studies on who connects with whom in various contexts. Voluminous and real-time social media data have been widely employed for epidemic surveillance or tracking emotional contagion [52,53]. A growing number of studies have employed user-generated content on social media to monitor emerging diseases at the breaking-out stage to minimize consequences or track trends in public health issues [54-56]. When public health scholars embrace new measures derived from social media data, empirically assessing and monitoring the quality of the new measures by cross-validating them with established measures is necessary. The parable of Google Flu Trends well illustrates the necessity of such cross-validation. When Google Flu Trends was first released, it outperformed traditional flu surveillance measures adopted by the US Centers for Disease Control and Prevention [57]. However, Google Flu Trends is reported to overestimate flu cases in the United States [58]. Validation of empirical measures is an ongoing process in public health research and beyond [59].

### When Public Health Research Meets Social Media: Unequal Status With Detached Concerns?

Social media–based public health research lies in the crossroad between public health studies and social science studies on information and communication technologies (ICTs) [60] and benefits from both perspectives. In the cooperative process, social media–based public health research reaches various levels in elaborating on the two perspectives. Taking the initial perspective of public health interest, many acceptability studies and randomized controlled trials have been documented to examine the effectiveness of social media to reach different public health goals [40,61]. In these studies, social media is often considered a new functional tool to improve public health. Meanwhile, in research that further examines the influence of ICTs on public health, who used what social media content targeted at whom through which social media platforms with what health effects is the core concern [62]. In this line of research, studies typically focus on the transmission of health information, communication between health agencies, the uses of health apps, and so on [63]. The inherent concerns of these studies seem to be detached though not in conflict in that social media facilitates the public health promotion process, and public health outcomes add value to the communication through social media.

From an overview of social media–based public health research, the dominating approach of these published studies considers public health issues as the substantial interests and ultimate outcomes rather than regard social media as an equally important area of concern. Many articles used limited space to describe the use of social media in health promotion campaigns or projects [64,65]. The subordinate role of social media suggests that the potential of ICTs has not been fully realized in the domain of public health [41]. Empirical studies should not only focus on what social media can contribute to public health research but should also examine how and why social media can make an impact in various contexts of public health research. This can substantially improve the understanding of the intended as well as unintended consequences social media can exert on health attitudes and behaviors. This can also enable public health researchers to integrate social media into their research design further.

### Limitations

Despite the strengths and contributions, this study has certain limitations. First, the study may suffer from the file drawer effect given that only studies indexed in Web of Science and PubMed were included. Empirical studies published in other outlets were not considered here. Future studies are warranted to expand the pools to conference proceedings and articles indexed in other databases. Second, this study used numerous diseases as search terms in the initial search, but the list remains incomplete. Some important diseases, such as mental disorders, were not incorporated. Despite this, the topic modeling captured mental health as a major theme. Further research is suggested to include mental health keywords as search terms. Third, LDA topic modeling is a well-recognized method to identify related themes through document-word matrices. However, the results of the topic modeling were not as neat as expected. No standard and quantitative thresholds exist for researchers to choose the optimal number of topics. Future studies are encouraged to replicate this study and examine the reliability of such themes.

### Conclusions

This study examined research themes, roles of social media, and research methods in social media–based public health research published from 2000 to 2018. This research identifies 25 research themes covering different diseases, various population groups, physical and mental health topics, and other significant issues. Social media assumes two major roles in public health research: one is to produce substantial research interest for public health research and the other is to furnish a research context for public health research. Social media enables scholars to study new phenomena and propose new research questions in public health research. Meanwhile, the methodological potential of social media in public health research needs further exploration.

## Appendix

Multimedia Appendix 1 Social media roles and definitions.

Multimedia Appendix 2 Network graph of topic-word probability of the 25 research themes.

Multimedia Appendix 3 Representative articles of the 25 research themes.

## Abbreviations

ICT: information and communication technology
LDA: latent Dirichlet allocation
MERS: Middle East respiratory syndrome
