# Supplementary material for: When Public Health Research Meets Social Media: Knowledge Mapping From 2000 to 2018
Source: J Med Internet Res. 2020 Aug 13;22(8):e17582. doi: 10.2196/17582 (PMC7453331; doi:10.2196/17582)
Supplement: Multimedia Appendix 1 [file jmir_v22i8e17582_app1.docx]

Supplementary Table 1 Social media roles and the definitions

| **Social Media Role** | |  | |  |
| --- | --- | --- | --- | --- |
| **1. Social media as substantial research interests** | | **Definition** | |  |
| Intervention | An interactive intervention tool targeted at changing personal and environmental risky health factors | Reduce health risk resulted from specific behaviors and attitudes through social media | | |
|  | An intervention information-distributing tool (one-way and not real-time interactive) | Regard social media as a channel to send interventions/alerts (e.g. SMS, etc.) | | |
|  | A source for health information seeking | Regard social media as a channel to receive health information | | |
|  | Usability test of social media platforms as intervention instruments | Focus on the characteristics/usability of health-related social media platforms. | | |
| Human-computer interaction characteristics | Public’s attitudes toward technology and social media in terms of health | Focus on the public’s attitudes towards technology and social media | | |
|  | Characteristics and behaviors of social media users and groups | Focus on the behavioral characteristics of specific social media users (e.g. vulnerable groups, patients, etc.) | | |
|  | Factors affecting users' health behavior or attitudes on social media | Focus on how social media affects users’ health | | |
|  | Consequences/influences on health behaviors caused by (popular) social media | Focus on what health results caused by social media | | |
| Social media as a platform of social influence | Building online (support) groups for patients | Focus on how patients interact with patients in social media |  |  |
|  | Facilitating physician–patient communication, or information seeker-provider communication | Focus on how doctors interact with patients in social media, or the interaction between health information seeker and provider |  |  |
|  | Enhancing health-related marketing | Focus on how health-related organizations or companies interact with the public in social media |  |  |
|  | Changing the health behavior of people all over the world at a macro level | Focus on the macro influence on people brought by the development of social media |  |  |
| Social media for disease surveillance, risk assessment or prevention | | Regard social media as a method to monitor, predict, and prevent disease |  |  |
| **2. Social media as research context** | |  |  |  |
| As a mere reference | | Regard social media as a research background or a research environment |  |  |
| As platforms for participant recruitment | Social media for participant recruitment | Regard social media as a channel to distribute questionnaires and recruit experiment participants |  |  |
|  | Social media as platforms to recruit their users | Regard social media as a channel to access to its own users |  |  |
| As a data source | Social media as text data source | Regard social media as a method to collect and analyze data |  |  |
|  | Social media as article search platforms for meta-analysis | Regard social media like Google as a channel to collect papers and conduct meta-analysis |  |  |
