# Supplementary figures and images for: When Public Health Research Meets Social Media: Knowledge Mapping From 2000 to 2018
Source: J Med Internet Res. 2020 Aug 13;22(8):e17582. doi: 10.2196/17582 (PMC7453331; doi:10.2196/17582)

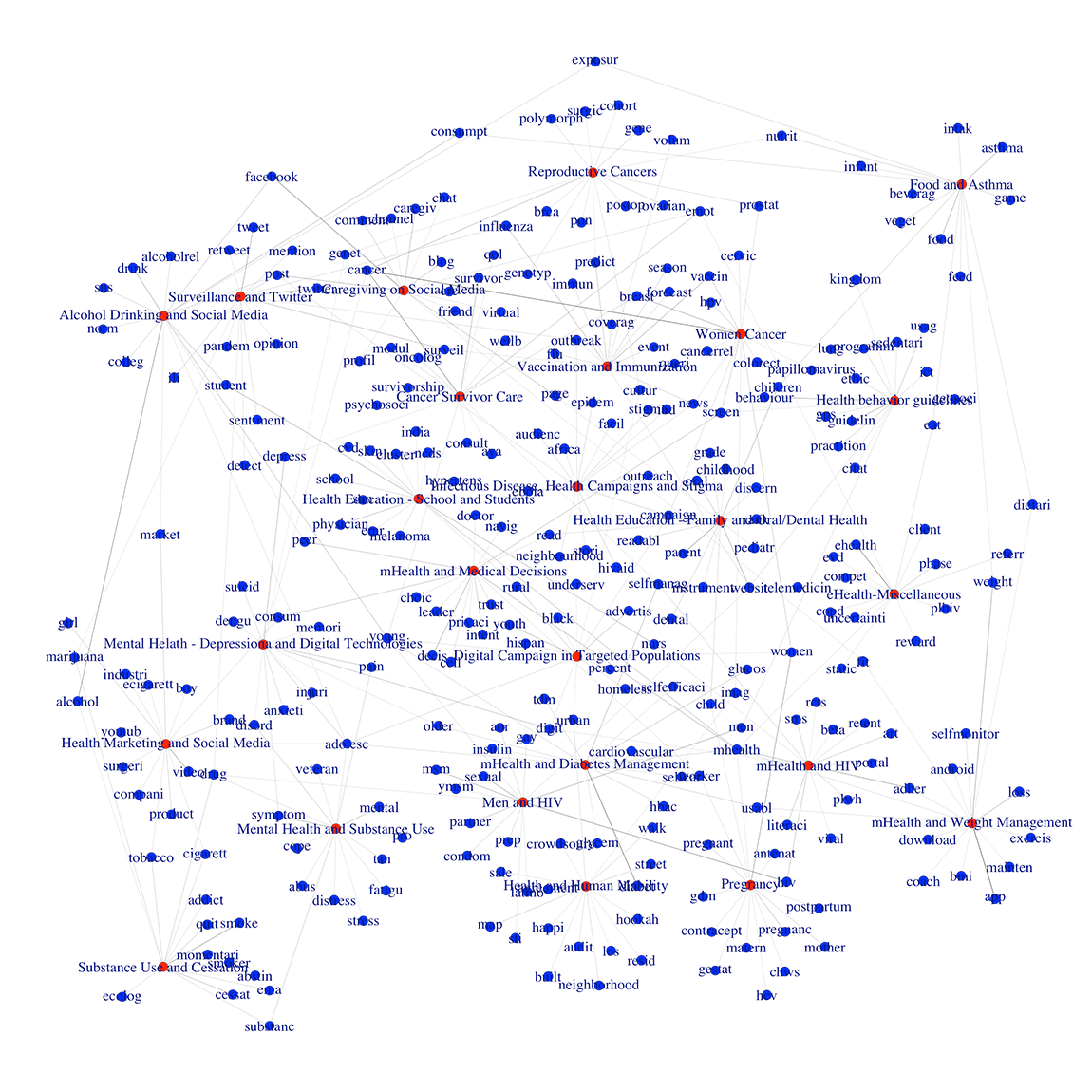

Supplement: Multimedia Appendix 2 [file jmir_v22i8e17582_app2.png]
